# Exfoliation in a low boiling point solvent and electrochemical applications of MoO_3_

**DOI:** 10.3762/bjnano.11.52

**Published:** 2020-04-17

**Authors:** Matangi Sricharan, Bikesh Gupta, Sreejesh Moolayadukkam, H S S Ramakrishna Matte

**Affiliations:** 1 Energy Materials Laboratory, Centre for Nano and Soft Matter Sciences, Jalahalli, Bengaluru 560013, India

**Keywords:** 2-butanone, liquid-phase exfoliation, low-boiling point solvent, molybdenum trioxide (MoO_3_), supercapacitors

## Abstract

MoO_3_ is a versatile two-dimensional transition metal oxide having applications in areas such as energy storage devices, electronic devices and catalysis. To efficiently utilize the properties of MoO_3_ arising from its two-dimensional nature exfoliation is necessary. In this work, the exfoliation of MoO_3_ is carried out in 2-butanone for the first time. The achieved concentration of the dispersion is about 0.57 mg·mL^−1^ with a yield of 5.7%, which are the highest values reported to date. These high values of concentration and yield can be attributed to a favorable matching of energies involved in exfoliation and stabilization of MoO_3_ nanosheets in 2-butanone. Interestingly, the MoO_3_ dispersion in 2-butanone retains its intrinsic nature even after exposure to sunlight for 24 h. The composites of MoO_3_ nanosheets were used as an electrode material for supercapacitors and showed a high specific capacitance of 201 F·g^−1^ in a three-electrode configuration at a scan rate of 50 mV·s^−1^.

## Introduction

The advent of graphene has opened a new area of research in the field of two-dimensional materials [1]. The extraordinary properties of graphene have led researchers to look into other layered materials, such as metal dichalcogenides (MoS_2_, WS_2_, WSe_2_), hexagonal boron nitride (h-BN), layered double hydroxides, metal hydroxides (Ni(OH)_2_, Co(OH)_2_), metal oxides (MoO_3_, WO_3_) and phyllosilicates, for various applications in different fields [2–5]. Among the layered materials, molybdenum oxide (MoO_3_) has gained special attention because of its numerous applications in electronics, catalysis, electrochemistry, solar cells and gas sensors [6]. Monolayered and few-layered MoO_3_ has been reported to have better properties than the bulk material [7]. Thus, it is crucial to exfoliate MoO_3_ for improved performance in a variety of applications.

Liquid-phase exfoliation (LPE) has been shown to be an effective technique for obtaining dispersions of two-dimensional materials. It also offers the advantages of low cost and scalability [2]. The LPE process is initiated either by ultrasonic vibrations or shearing in a liquid medium that facilitates the exfoliation. To influence the energies relevant for the exfoliation in aqueous media, additives such as surfactants and polymers are used [8]. However, their removal is quite tedious and the remnants are detrimental for some applications. To alleviate this, LPE has been carried out in organic solvents taking into account the Hansen solubility parameters (HSPs) [9]. To exfoliate MoO_3_ in organic solvents without any additives, Coleman et al. have found that *N*-methyl-2-pyrrolidone (NMP), *N*-cyclohexyl-2-pyrrolidone, and dimethylformamide are the best solvents [10]. The high boiling point of these dispersions (bp > 150 °C) restrict the usage in flexible devices along with issue that these solvents are also toxic (NFPA 704 health code above 2 [11]).

There are reports in literature where MoO_3_ has been exfoliated in solvents with low boiling point, such as isopropyl alcohol (IPA) [5]. Alternative approaches for obtaining MoO_3_ dispersions in low boiling point solvents have also been reported. For instance, Alsaif et al. have exfoliated MoO_3_ in mixtures of water and alcohols (methanol, ethanol, IPA) [12]. In these dispersions molybdenum bronze (H*_x_*MoO_3_) was formed after exposure to UV radiation, making MoO_3_ quasi-metallic rather than semiconducting [13–14]. Thus, it is of highest priority to produce MoO_3_ dispersions of high concentrations and yields while maintaining the semiconductor properties of MoO_3_.

In an attempt to address the aforementioned issues, we exfoliated MoO_3_ in 2-butanone, an environmentally benign solvent (NFPA 704 health code = 1) with low boiling point (bp = 80 °C) for the first time. We obtained MoO_3_ concentrations up to 0.57 mg·mL^−1^ with a yield of 5.7%. It is noteworthy that, the chemical nature of the MoO_3_ dispersions was not altered after exposure to sunlight (UV radiation) for 24 h. The exfoliated MoO_3_ was used as electrode material for supercapacitor applications. The specific capacitance values were as high as 221 F·g^−1^ at 5 mV·s^−1^ with good rate capability and capacitance retention in a three-electrode system.

## Experimental

### Liquid-phase exfoliation of MoO_3_

Bulk molybdenum trioxide (MoO_3_) powder was purchased from Sigma-Aldrich (99% purity, 300 mesh) and 2-butanone was procured from Finar Limited (AR, 98% purity). All materials were used without further purification. Bulk MoO_3_ powder suspensions with different initial concentrations of 5, 7.5, 10, and 20 mg·mL^−1^ were sonicated with a probe sonicator Vibra cell VCX 750. All exfoliations were carried out at 60% amplitude using a 13 mm diameter horn in a 100 mL cooling cell maintaining the temperature below 20 °C. For comparison, exfoliations in IPA and IPA/H_2_O (1:1) were also performed using a similar protocol. After sonication, the dispersions were allowed to rest for 12 h before centrifuging at 500 rpm (REMI Neya-12) for 1 h to remove non-exfoliated flakes. Small aliquots were collected at regular time intervals in order to measure the concentration. The absorbance of the MoO_3_ dispersions was recorded using a UV–vis spectrophotometer (Perkin Elmer Lambda 750) in 10 mm quartz cuvettes. The concentrations of dispersions were determined by using thermogravimetric analysis. For this, 4 mL of MoO_3_ dispersion was filled in a 5 mL beaker followed by drying off the solvent at 80 °C in a preheated oven. The remaining MoO_3_ powder in the beaker was weighed to determine the concentration. The morphology of MoO_3_ flakes was characterized using field-emission scanning electron microscopy (FESEM; Tescan Mira3), transmission electron microscopy (TEM; FEI Talos, 200 kV) and atomic force microsopy (AFM; Agilent 5500). Samples for FESEM and AFM were prepared by dripping 10 µL of MoO_3_ dispersion (diluted 100 times) onto a Si/SiO_2_ (300 nm) substrate while samples for TEM were prepared by dripping 10 µL of the diluted dispersion on a 300 mesh lacey carbon grid. Raman spectra (Horiba LABRam HR) of the MoO_3_ layers were recorded using a 532 nm excitation laser. X-ray diffractograms (XRD; Rigaku Smart lab) of the bulk and exfoliated MoO_3_ were obtained using a Cu Kα (1.54 Å) X-ray source. The surface potential of MoO_3_ dispersions was determined by zeta potential measurements using a Malvern Zetasizer NanoZS. All electrochemical measurements were carried out using Autolab PGSTAT302N.

#### Electrode preparation and electrochemical testing

**Three-electrode system:** A glassy carbon electrode (GCE, 0.3 cm diameter) as the working electrode, Pt wire as counter electrode and a saturated calomel electrode (SCE) as reference electrode were used for the electrochemical testing of the exfoliated MoO_3_ dispersions and its composites. In brief, the GCE was cleaned with a polishing cloth using fine alumina abrasive powders and washed thoroughly in deionized water. The required amount of the dispersion of known concentration was dripped onto the cleaned GCE using a micropipette and dried under ambient conditions. Nafion® was used as the binder. To study the effect of the conducting additive, different ratios of conductive carbon black (CB) were added and sonicated for 15 min to obtain homogeneous dispersions, which were then dripped on the GCE. All electrochemical measurements were performed in 1 M H_2_SO_4_ electrolyte.

**Two-electrode system:** The optimized ratio obtained from measurements of the three-electrode configuration were used to fabricate two-electrode devices. The respective amounts of MoO_3_ and carbon black were mixed with 5 wt % of PVDF and stirred overnight in NMP to form a thick paste. The paste was used to make a thin electrode film on carbon paper (1.5 cm × 1.5 cm) and dried in an oven at 60 °C. To fabricate two-electrode supercapacitors, two such electrodes were sandwiched between battery-grade steel current collectors, separated by filter paper dipped in 1 M H_2_SO_4_ electrolyte.

## Results and Discussion

**Figure 1:**
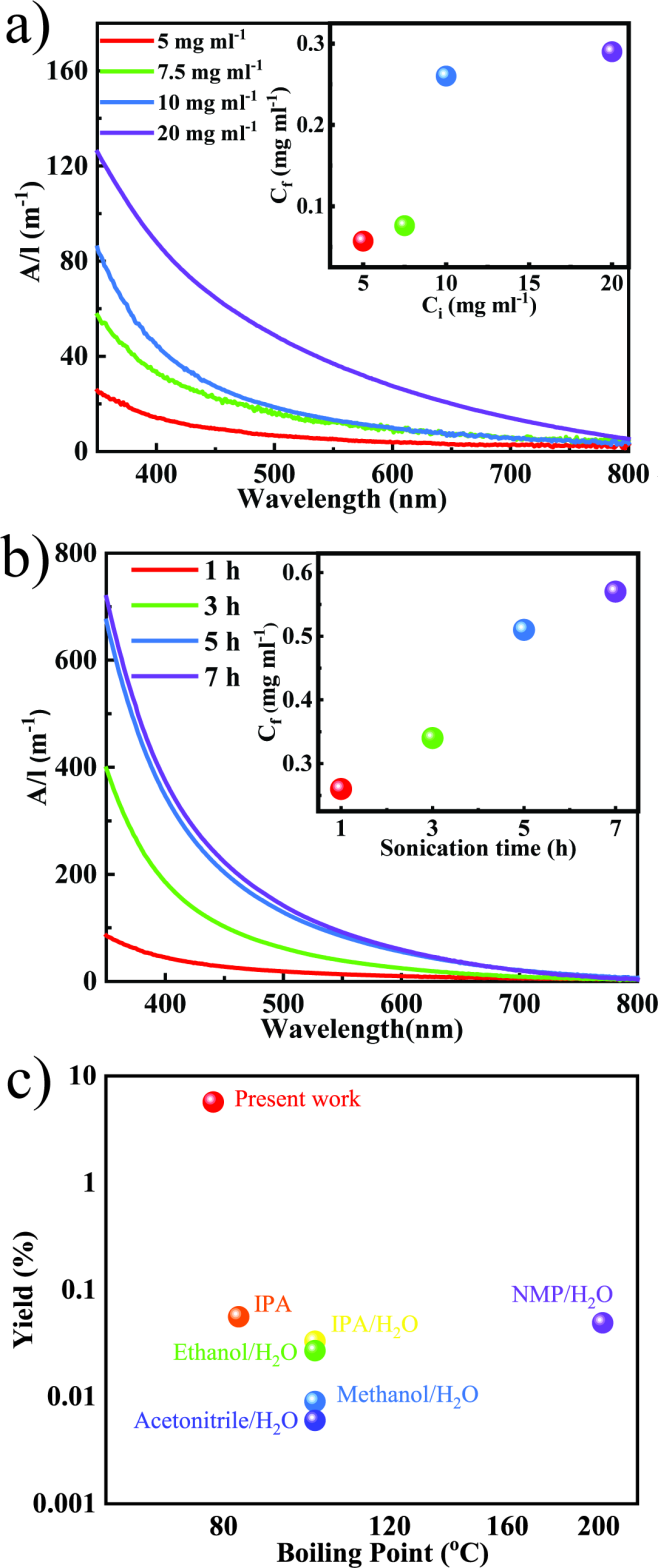
(a) UV–vis spectra of MoO_3_ dispersions obtained from different initial concentrations (*C*_i_). The inset shows the final concentration as a function of the initial concentration; (b) UV–vis spectra of MoO_3_ dispersions obtained from *C*_i_ = 10 mg·mL^−1^ after different sonication durations. The inset shows the final concentration as a function of the sonication time; (c) comparison of previously reported MoO_3_ dispersion yields in different solvents [5,12,21].

LPE assisted by tip sonication is an effective technique to peel off mono- and few-layers from layered bulk materials. In the crystal structure of α-MoO_3_ the atoms are connected to layers through distorted edge- and corner-sharing MoO_6_ octahedra. The layers are linked through weak out-of-plane van der Waals interactions. For exfoliation of MoO_3_, 2-butanone, a low boiling point solvent, was chosen the HSP values of which match well with those of MoO_3_ (Supporting Information File 1, Table S1). To study the exfoliation efficiency, different initial concentrations (*C*_i_) of MoO_3_ (5, 7.5, 10 and 20 mg·mL^−1^) were sonicated for 1 h using probe sonication. The UV–vis spectra of the dispersions were collected and the absorbance per unit length (*A*/*l*) is shown in Figure 1a. As *C*_i_ of the dispersion increases the *A*/*l* value also increases. This suggest that with an increase of *C*_i_, the final concentration (*C*_f_) of the dispersion also increases. This is probably due to the larger amount of material available for exfoliation. The inset in Figure 1a shows the *C*_f_ of the dispersions as a function of *C*_i_. To understand the impact of the sonication time on *C*_f_, MoO_3_ with *C*_i_ = 10 mg·mL^−1^ (time-dependent studies with other *C*_i_ values are shown in Figure S1, Supporting Information File 1) was sonicated for different periods of time (1, 3, 5, and 7 h). The UV–vis spectra of the dispersions were recorded and are shown in Figure 1c. From Figure 1c, it is evident that as the time of sonication increases the *A*/*l* value increases, which indicates that *C*_f_ increases with increase in sonication time (a similar trend is also observed for other *C*_i_ values (Figure S1, Supporting Information File 1). The maximum *C*_f_ of 0.57 mg·mL^−1^ (shown in inset of Figure 1b) with a yield of 5.7% is achieved after sonication for 7 h. It is worth noting that this is the highest concentration and yield of MoO_3_ dispersions achieved to date, to the best of our knowledge (Figure 1c). The high concentration and yield can be attributed to a favorable matching of the exfoliation and stabilization energies between the solvent and the MoO_3_ nanosheets. It has been theoretically reported that apart from the matching of HSP values, factors such as the structure of the solvent, its bulkiness and its re-orientation on the exfoliated nanosheets are other critical parameters to be considered for efficient exfoliation [15–16]. These theoretical studies were also supplemented with experimental observations where it was demonstrated that the simple addition of a –CH_2_ group to a solvent drastically changes the efficiency of the LPE process [17–19]. It is also reported that the exfoliation efficiency of layered materials may differ depending on the bulk precursor. To validate this, the exfoliation of MoO_3_ was carried out from two different precursors procured from different manufacturers. Similar concentrations of MoO_3_ dispersions were obtained under identical experimental conditions (Supporting Information File 1, Figure S2) [20].

**Figure 2:**
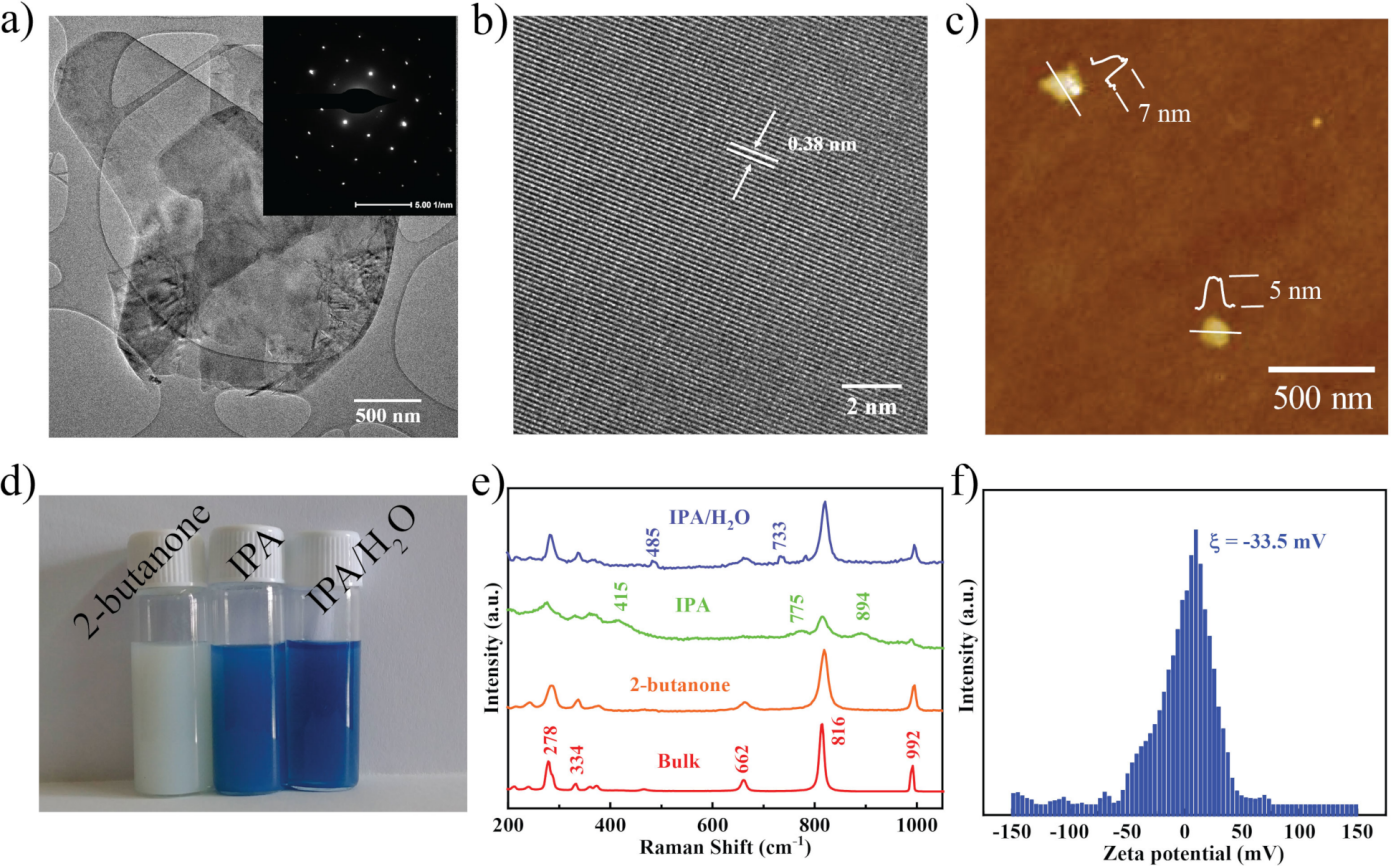
(a) TEM micrograph of MoO_3_ nanosheets. The inset shows the SAED pattern; (b) HRTEM micrograph of MoO_3_ nanosheets; (c) AFM micrograph of MoO_3_ nanosheets; (d) photograph of MoO_3_ dispersions in 2-butanone, IPA and IPA/H_2_O mixture; (e) Raman spectra of bulk and exfoliated MoO_3_ in different solvents; (f) zeta potential of MoO_3_ dispersions in 2-butanone.

The structural and chemical nature of the exfoliated MoO_3_ nanosheets was determined using microscopic and spectroscopic techniques. The two-dimensional nature of MoO_3_ is demonstrated in the TEM micrograph in Figure 2a. Supporting Information File 1, Figure S3c,d, also shows ultrathin nanosheets of MoO_3_ suggesting a successful exfoliation. The selected-area electron diffraction (SAED) pattern shown in the inset of Figure 2a indicates that the MoO_3_ nanosheets are crystalline after exfoliation. Crystallinity and retention of the orthorhombic phase of exfoliated MoO_3_ nanosheets are evident from XRD (Figure S4, Supporting Information File 1). The HRTEM micrograph in Figure 2b shows a *d*-spacing of 0.38 nm corresponding to the (110) planes of orthorhombic MoO_3_ (indexed with JCPDS file No. 05-0506). The AFM micrograph in Figure 2c shows the topography of MoO_3_ nanosheets the thickness values of which suggest the presence of 5–7 layers [6]. The FESEM micrographs shown in Supporting Information File 1 corroborate the exfoliation of MoO_3_. Figure S3a,b (Supporting Information File 1) shows bulk MoO_3_ and exfoliated nanosheets of MoO_3_, respectively.

Along with the morphological characterization it is also important to assess the chemical nature of the exfoliated nanosheets. It was previously observed that the exfoliation of MoO_3_ in some low boiling point alcohols (IPA, ethanol) and mixed solvent systems based on H_2_O (IPA/H_2_O, ethanol/H_2_O) tend to lead to the formation of molybdenum bronze (H*_x_*MoO_3_) when exposed to UV radiation [12,21]. The mechanism of the reaction is [13]:


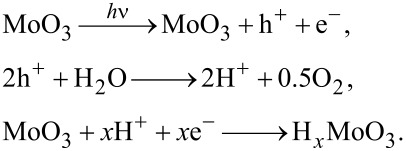


H^+^ ions, which are dissociated from the protic solvents, tend to intercalate in MoO_3_ layers forming H*_x_*MoO_3_ and the MoO_3_ dispersions change the color to blue. It is worth mentioning that exfoliation in 2-butanone did not result in any color change when exposed to sunlight (UV radiation) for 24 h unlike IPA and IPA/H_2_O mixture (Figure 2d). The probable reason is the aprotic nature of 2-butanone, which does not support the formation of H^+^ in the presence of UV light. This was further confirmed using Raman spectroscopy and UV–vis spectroscopy (Supporting Information File 1, Figure S5). The Raman spectra of bulk and exfoliated MoO_3_ in 2-butanone, IPA and an IPA/H_2_O mixture are shown in Figure 2e. Bulk and exfoliated MoO_3_ in 2-butanone has strong Raman peaks at 278, 334, 662, 816 and 992 cm^−1^, which are in good agreement with orthorhombic α-MoO_3_, suggesting that the exfoliated MoO_3_ retained its chemical structure [13]. Additionally, the increase in full width at half maximum (FWHM) from 4 cm^−1^ (bulk) to 8 cm^−1^ (exfoliated in 2-butanone) confirms the exfoliation of MoO_3_. However, MoO_3_ exfoliated in IPA shows additional peaks at 415, 775 and 894 cm^−1^, which are due to a deformation of Mo–O bonds confirming the formation of H*_x_*MoO_3_ [13]. Similarly, the MoO_3_ nanosheets exfoliated in the IPA/H_2_O mixture show the evolution of some new peaks at 485 and 733 cm^−1^ suggesting the presence of MoO_3−_*_x_* [13]. The Raman spectra clearly suggest that MoO_3_ nanosheets are chemically stable in 2-butanone even after exposure to sunlight. Zeta potential measurements were carried out to determine the charge on the surface of the nanosheets, which is critical for the stability of the dispersions. The high zeta potential of −33.5 mV (Figure 2f) affirms the stability of MoO_3_ dispersions in 2-butanone.

**Figure 3:**
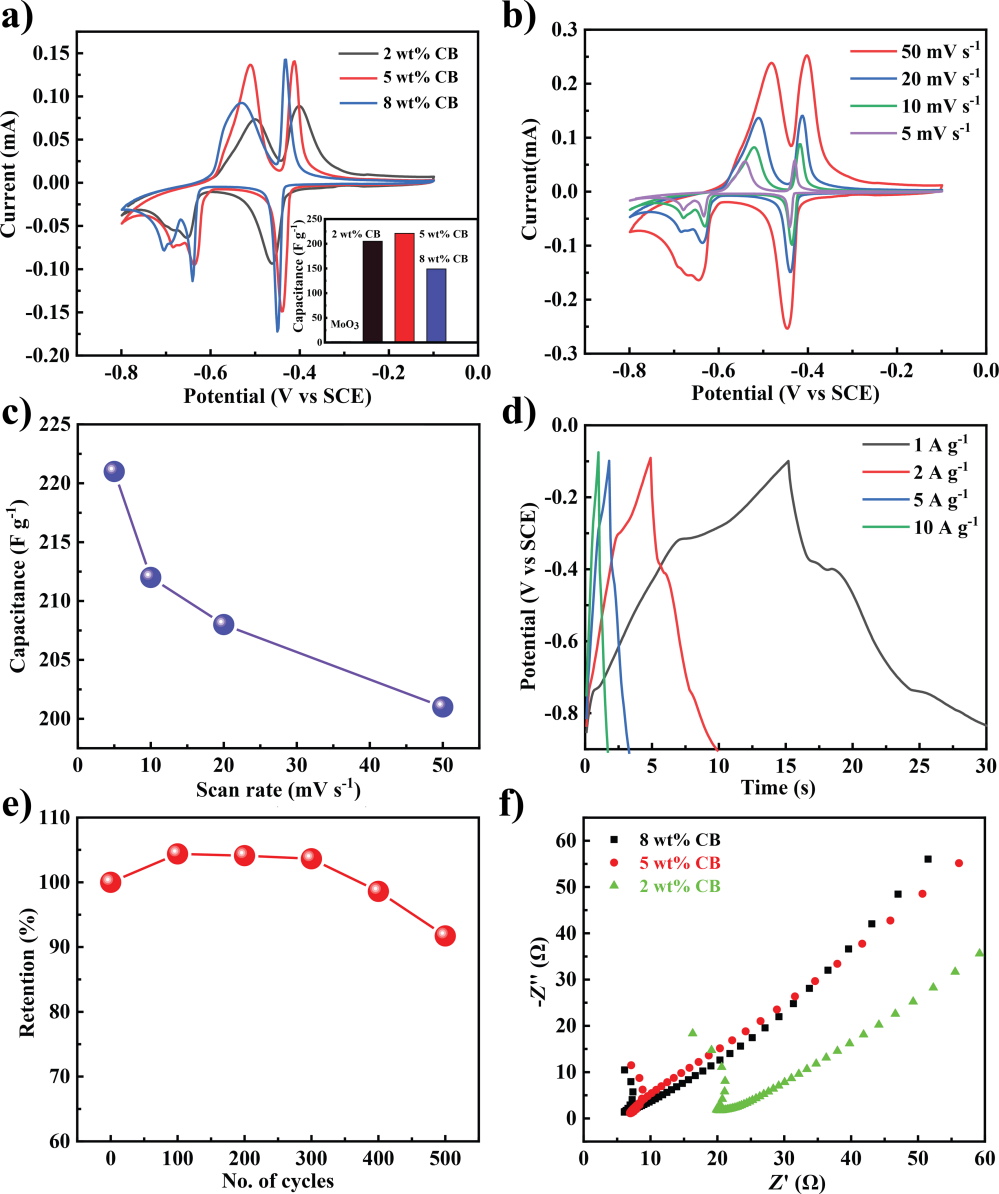
(a) CV measurement of MoO_3_/carbon black composites showing pseudo capacitive behavior, inset shows the change in capacitance with increasing mass fraction of carbon black; (b) performance of a composite electrode with 5 wt % CB as a function of the changing scan rate; (c) capacitance as a function of the scan rate, (d) galvanostatic charge–discharge profile of the 5 wt % CB composite; (e) capacitance retention of the 5 wt % CB composite; (f) EIS of the composites.

Exfoliated two-dimensional materials are known to exhibit good electrochemical properties compared to the bulk materials [22–23]. The electrochemical properties of the exfoliated MoO_3_ nanosheets were evaluated using a three-electrode configuration and are shown in Figure 3. Figure 3a shows the cyclic voltammetry (CV) measurement of the electrodes recorded between −0.8 and −0.1 V with a scan rate of 50 mV/s. Initially, pristine exfoliated MoO_3_ sheets were studied regarding the charge-storage properties. But, the pristine MoO_3_ nanosheets did not show any appreciable currents associated with redox peaks and the calculated specific capacitance was found to be very low (around 2 F·g^−1^, Supporting Information File 1, Figure S6). This may be attributed to the poor intrinsic electronic conductivity of the MoO_3_ [24]. In order to enhance the electrochemical properties, composites of exfoliated MoO_3_ nanosheets and conducting carbon black (CB) were prepared. As shown in Figure 3a, well-defined oxidation and reduction peaks are observed after the addition of 2 wt % CB. The two sets of redox peaks at −0.38 V/−0.47 V and −0.47 V/−0.66 V correspond to the reversible intercalation of the H^+^ ions into the MoO_3_ layers [25].

In addition, composites were prepared by adding 5 and 8 wt % of CB, and the corresponding CV measurements are shown in Figure 3a. The highest specific capacitance was observed for the composite that contains 5 wt % CB with values reaching up to 221 F·g^−1^. The composites with 2 and 8 wt % CB show specific capacitance values of 205 and 149 F·g^−1^, respectively, at a scan rate of 5 mV·s^−1^. The optimum amount of CB to make conductive pathways in MoO_3_ appears to be 5 wt % [26–27]. The change in specific capacitance as a function of the amount of CB is shown in the inset of Figure 3a. The rate capabilities can be understood based on the change in performance as a function of the scan rate [28]. To study this, the composite with 5 wt % CB was cycled at different scan rates (Figure 3b). The change in specific capacitance as a function of the scan rate is shown in Figure 3c. Even at a high scan rate of 50 mV·s^−1^ the electrode retained a capacitance value of 200 F·g^−1^ suggesting that it is a suitable material for fast-charging applications. This property could be assigned to the two-dimensional nature of the MoO_3_ nanosheets, which possess a high surface area allowing for almost unhampered diffusion and electrochemical interaction [29]. To study the effect of exfoliation on the electrochemical properties, bulk MoO_3_ with 5 wt % of CB was fabricated and tested under similar conditions. Compared to composites with exfoliated MoO_3_ the performance of the composite with bulk MoO_3_ is worse (Supporting Information File 1, Figure S6). A comparison of the performance of MoO_3_-based supercapacitors is shown in Table S2 (Supporting Information File 1). Galvanostatic charge–discharge measurements are a method to study the charge–discharge characteristics of electrode materials. The composite with 5 wt % CB was charged and discharged at different current densities and pseudocapacitive behavior was observed (Figure 3d). The charge–discharge profile shows a change in slope, which could be due to the electrochemically reversible hydrogen intercalation, which was also seen in the voltammetry curves. The charge and discharge times were found to be 15 and 13 s at a current density of 1 A·g^−1^. Cycling stability is a key factor for the commercialization of supercapacitors [30–31]. The composite with 5 wt % CB was tested for about 500 cycles (Figure 3e). Initially the specific capacitance was found to increase, which can be attributed to the wetting of the active material in the initial cycles [26]. Electrochemical impedance spectroscopy was used to study the effect of CB (Figure 3f). The addition of carbon black leads to a reduction of charge transfer resistance in the composites with 5 and 8 wt % CB (6 Ω) compared to the composite with 2 wt % CB (19 Ω). The charge–transfer curve is similar for 5 and 8 wt % CB, which implies that the effect of the additive saturates at 5 wt % CB. This observation also supports the observation from voltammetry data where the composite with 5 wt % CB shows a better performance.

**Figure 4:**
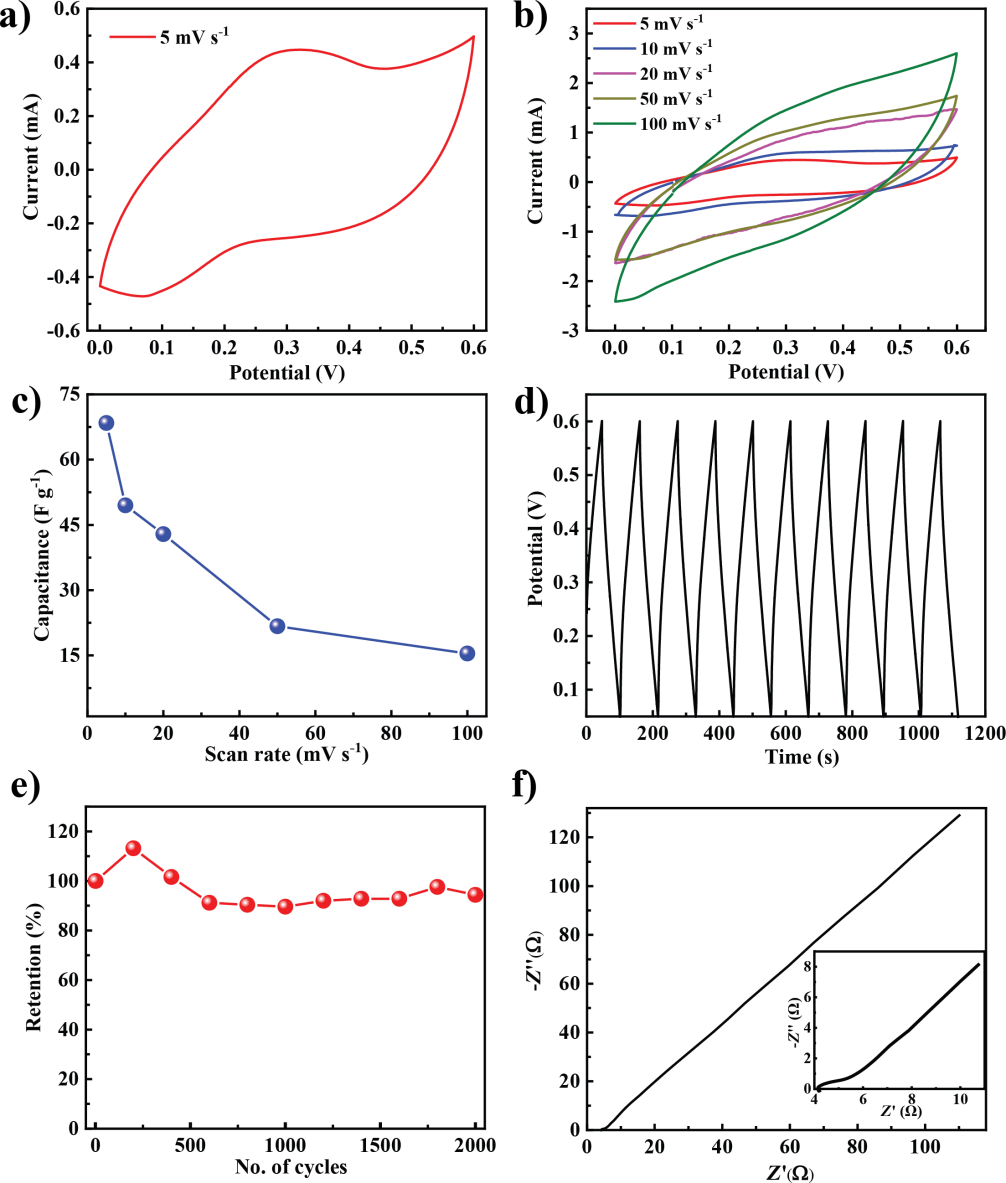
Supercapacitor characterization of a MoO_3_/5 wt % CB composite in two-electrode configuration. (a) CV curve of the symmetric capacitor at a scan rate 5 mV·s^−1^; (b) CV curve at different scan rates; (c) capacitance as a function of the scan rate; (d) galvanostatic charge–discharge; (e) cyclic stability and (f) electrochemical impedance spectrum with enlarged view in the inset.

Typically, electrode materials are tested for supercapacitor applications in a three-electrode configuration. But for practical applications, it is appropriate to test them in a two-electrode configuration. The optimized material from the three-electrode system (MoO_3_/5 wt % CB composite) was chosen for fabricating a two-electrode supercapacitor device (Figure 4). The CV curve of the capacitor at a scan rate of 5 mV·s^−1^ is shown in Figure 4a in a potential window from 0 to 0.6 V. The humps indicating a pseudocapacitance may be attributed to redox reactions in MoO_3_ as discussed for the three-electrode measurements. CV measurements have been carried out at different scan rates (Figure 4b). A maximum specific capacitance of 68.4 F·g^−1^ at a scan rate of 5 mV·s^−1^ was obtained. The capacitor also shows good rate capabilities (Figure 4c). The specific capacitance values varied from 18 to 68 F·g^−1^ at scan rates between 50 and 5 mV·s^−1^.

Charge–discharge characteristics of the capacitor are shown in Figure 4d. It shows a capacitive charge–discharge response with a small *iR* drop. This can be attributed to the better conductivity after adding CB. To study the stability of the composite, cyclic voltammetry was carried out up to 2000 cycles. Initially, the capacitance increases, which may be attributed to the wetting of the electrode with the electrolyte and the activation of available sites. Also, the composite shows a capacitance retention up to 94% even after 2000 cycles. Electrochemical impedance spectroscopy (EIS) is used to study the electrochemical series resistance and ideal nature of the capacitor. As shown in Figure 4f, EIS shows a typical plot having a semi-circular loop followed by a straight line. The magnified view in the inset of Figure 4f shows that the composite with 5 wt % CB has a low internal resistance, supporting the observation from the charge–discharge curve.

## Conclusion

The exfoliation of MoO_3_, carried out in the low boiling point solvent 2-butanone using probe sonication, resulted in a concentration of 0.57 mg·mL^−1^ and a yield of 5.7%, which are the highest values reported to date. Additionally, the MoO_3_ dispersions in 2-butanone do not undergo any chemical transformation when exposed to sunlight. Composites of the exfoliated MoO_3_ nanosheets with carbon black show a high specific capacitance of 201 F·g^−1^ at 50 mV·s^−1^ in a three-electrode configuration. In a two-electrode configuration, the electrode shows a high stability for 2000 cycles with 94% capacitance retention. We believe the process reported here can be used for the fabrication of flexible supercapacitors for wearable electronics.

## Supporting Information

File 1 Additional experimental data.

## References

[1] Geim A K, Novoselov K S, Rodgers P (2010). The rise of graphene. Nanoscience and Technology: A Collection of Reviews from Nature Journals.

[2] Nicolosi V, Chhowalla M, Kanatzidis M G, Strano M S, Coleman J N (2013). Science.

[3] Harvey A, He X, Godwin I J, Backes C, McAteer D, Berner N C, McEvoy N, Ferguson A, Shmeliov A, Lyons M E G (2016). J Mater Chem A.

[4] McAteer D, Godwin I J, Ling Z, Harvey A, He L, Boland C S, Vega-Mayoral V, Szydłowska B, Rovetta A A, Backes C (2018). Adv Energy Mater.

[5] Mendoza-Sánchez B, Hanlon D, Coelho J, O’Brien S, Pettersson H, Coleman J, Nicolosi V (2016). 2D Mater.

[6] de Castro I A, Datta R S, Ou J Z, Castellanos-Gomez A, Sriram S, Daeneke T, Kalantar-zadeh K (2017). Adv Mater (Weinheim, Ger).

[7] Quek S Y, Biener M M, Biener J, Friend C M, Kaxiras E (2005). Surf Sci.

[8] Coleman J N (2009). Adv Funct Mater.

[9] Coleman J N, Lotya M, O'Neill A, Bergin S D, King P J, Khan U, Young K, Gaucher A, De S, Smith R J (2011). Science.

[10] Hanlon D, Backes C, Higgins T M, Hughes M, O’Neill A, King P, McEvoy N, Duesberg G S, Mendoza Sanchez B, Pettersson H (2014). Chem Mater.

[11] National Fire Protection Association (2011). NFPA 704, Standard System for the Identification of the Hazards of Materials for Emergency Response.

[12] Alsaif M M Y A, Field M R, Daeneke T, Chrimes A F, Zhang W, Carey B J, Berean K J, Walia S, van Embden J, Zhang B (2016). ACS Appl Mater Interfaces.

[13] Alsaif M M Y A, Latham K, Field M R, Yao D D, Medehkar N V, Beane G A, Kaner R B, Russo S P, Ou J Z, Kalantar-zadeh K (2014). Adv Mater (Weinheim, Ger).

[14] Inzani K, Nematollahi M, Vullum-Bruer F, Grande T, Reenaas T W, Selbach S M (2017). Phys Chem Chem Phys.

[15] Sresht V, Pádua A A H, Blankschtein D (2015). ACS Nano.

[16] Khan U, Porwal H, O’Neill A, Nawaz K, May P, Coleman J N (2011). Langmuir.

[17] Lobo K, Trivedi S, Matte H S S R (2019). Nanoscale.

[18] Gupta B, Matte H S S R (2019). ACS Appl Electron Mater.

[19] Halim U, Zheng C R, Chen Y, Lin Z, Jiang S, Cheng R, Huang Y, Duan X (2013). Nat Commun.

[20] Shen J, Wu J, Wang M, Dong P, Xu J, Li X, Zhang X, Yuan J, Wang X, Ye M (2016). Small.

[21] Razmyar S, Sheng T, Akter M, Zhang H (2019). ACS Appl Nano Mater.

[22] Sreejesh M, Huang N M, Nagaraja H S (2015). Electrochim Acta.

[23] Wu S, Zeng Z, He Q, Wang Z, Wang S J, Du Y, Yin Z, Sun X, Chen W, Zhang H (2012). Small.

[24] Chang J, Jin M, Yao F, Kim T H, Le V T, Yue H, Gunes F, Li B, Ghosh A, Xie S (2013). Adv Funct Mater.

[25] Tang W, Liu L, Tian S, Li L, Yue Y, Wu Y, Zhu K (2011). Chem Commun.

[26] Sreejesh M, Dhanush S, Rossignol F, Nagaraja H S (2017). Ceram Int.

[27] Shanbhag D, Bindu K, Aarathy A R, Ramesh M, Sreejesh M, Nagaraja H S (2017). Mater Today Energy.

[28] Zhang L, Shi G (2011). J Phys Chem C.

[29] Zhou K, Zhou W, Liu X, Sang Y, Ji S, Li W, Lu J, Li L, Niu W, Liu H (2015). Nano Energy.

[30] Sreedhara M B, Matte H S S R, Govindaraj A, Rao C N R (2013). Chem – Asian J.

[31] Zhou J, Lian J, Hou L, Zhang J, Gou H, Xia M, Zhao Y, Strobel T A, Tao L, Gao F (2015). Nat Commun.

