# Supplementary material for: Exfoliation in a low boiling point solvent and electrochemical applications of MoO3
Source: Beilstein J Nanotechnol. 2020 Apr 17;11:662–70. doi: 10.3762/bjnano.11.52 (PMC7176920; doi:10.3762/bjnano.11.52)
Supplement: File 1 — Additional experimental data. [file Beilstein_J_Nanotechnol-11-662-s001.pdf]

BEILSTEIN JOURNAL OF NANOTECHNOLOGY

Supporting Information

for

Exfoliation in a low boiling point solvent and electrochemical applications of MoO_3

Matangi Sricharan, Bikesh Gupta, Sreejesh Moolayadukkam
and H. S. S. Ramakrishna Matte

Beilstein J. Nanotechnol. **2020,** *11,* 662–670. doi:10.3762/bjnano.11.52

Additional experimental data

License and Terms: This is a supporting information file under the terms of the Creative Commons Attribution License (http://creativecommons.org/licenses/by/4.0). Please note that the reuse, redistribution and reproduction in particular requires that the authors and source are credited.
The license is subject to the *Beilstein Journal of Nanotechnology* terms and conditions: (https://www.beilstein-journals.org/bjnano)

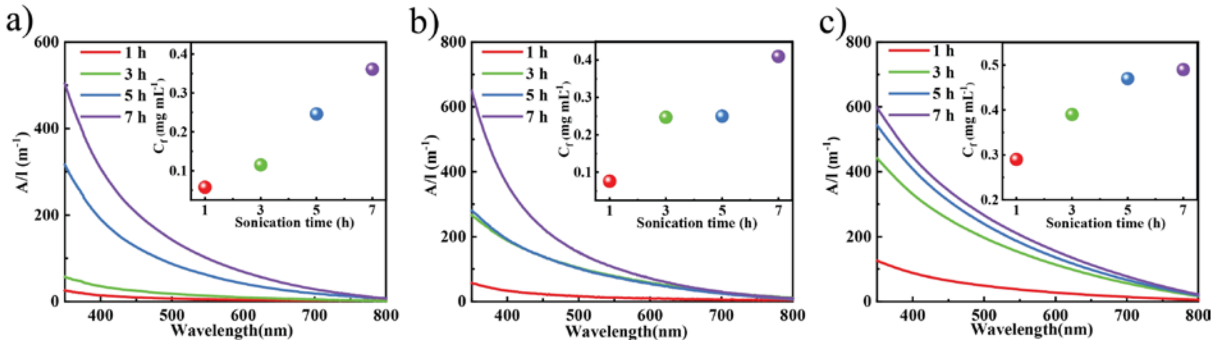

Figure S1: UV–vis spectra of MoO_3 dispersions obtained from C_i of (a) 5 mg·mL^{-1} (b) 7.5 mg·mL^{-1} and (c) 20 mg·mL^{-1}.

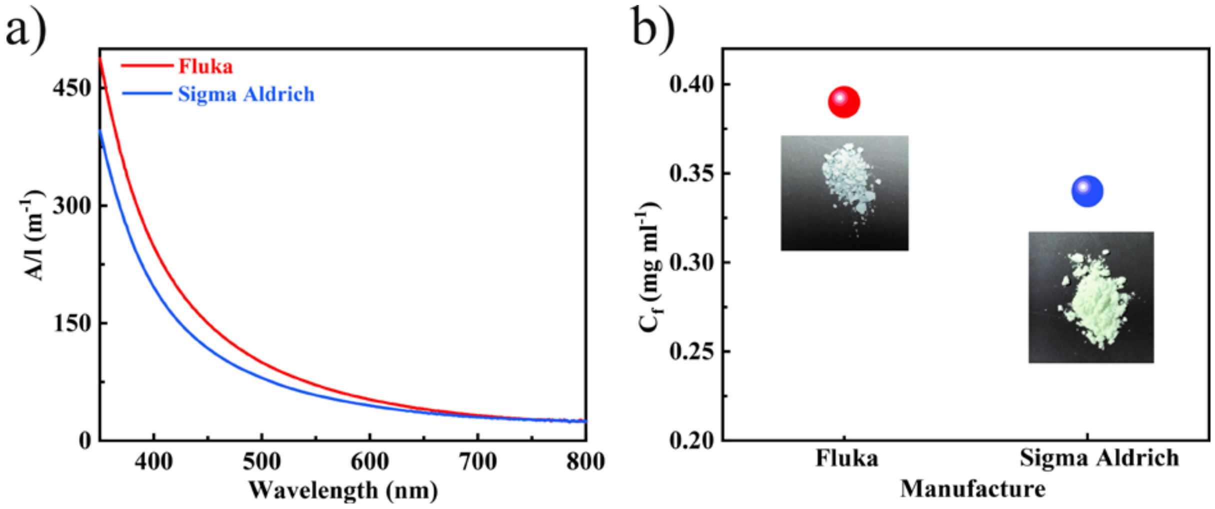

Figure S2: (a) UV–vis spectra of MoO_3 dispersions with MoO_3 procured from two different sources (Fluka and Sigma-Aldrich). MoO_3 exfoliation was carried out in 2-butanone with C_i of 10 mg·mL^{-1} and 3 h of sonication. (b) C_f of MoO_3 dispersions obtained from different manufacturers. Insets show digital micrographs of the MoO_3 powders obtained from different manufacturers.

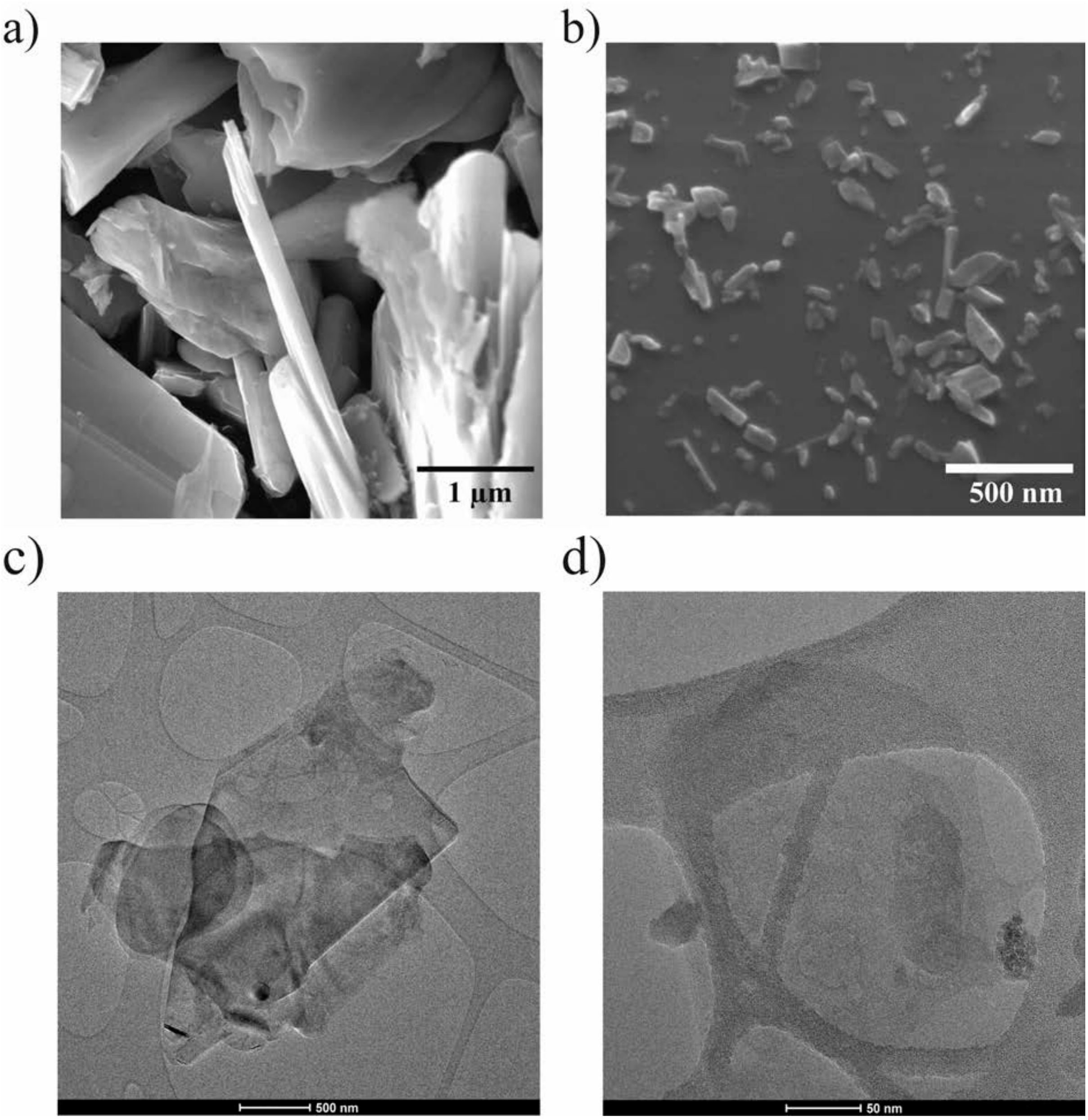

Figure S3: FESEM micrographs of (a) bulk and (b) exfoliated MoO_3; (c, d) TEM micrographs of exfoliated MoO_3 nanosheets.

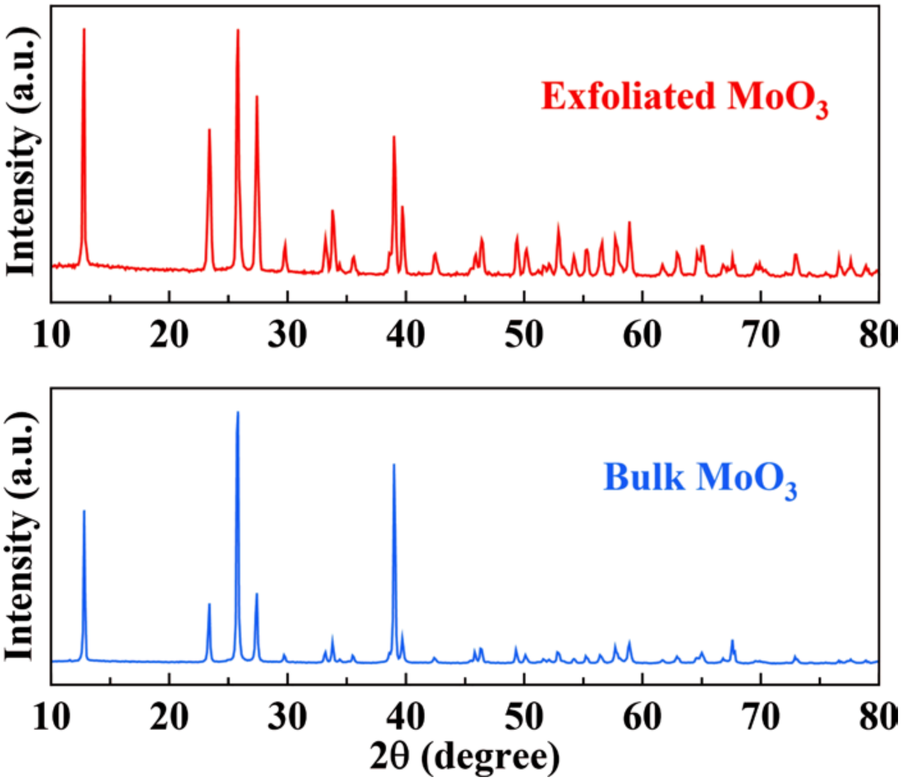

Figure S4: XRD of bulk and exfoliated MoO_3.

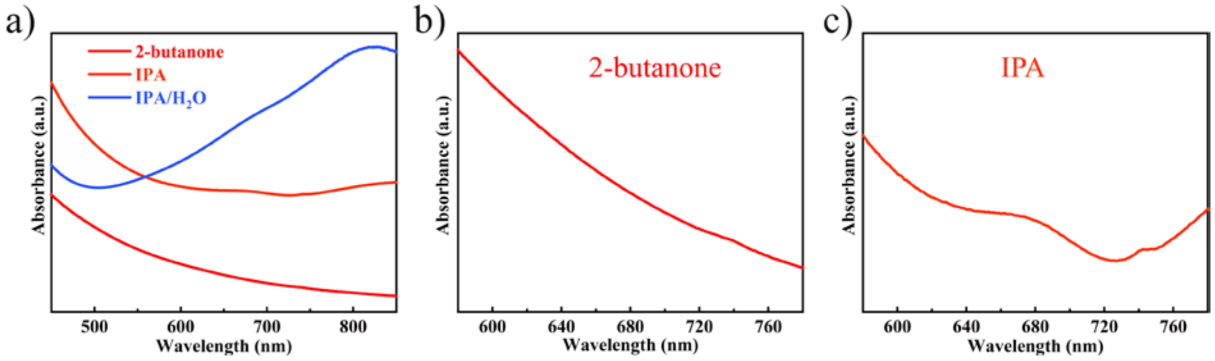

Figure S5: (a) UV–vis spectra of MoO_3 in 2-butanone, IPA and IPA/H_2O. (b) Enlarged view of the UV–vis spectrum in 2-butanone. (c) Enlarged view of the UV–vis spectrum in IPA.

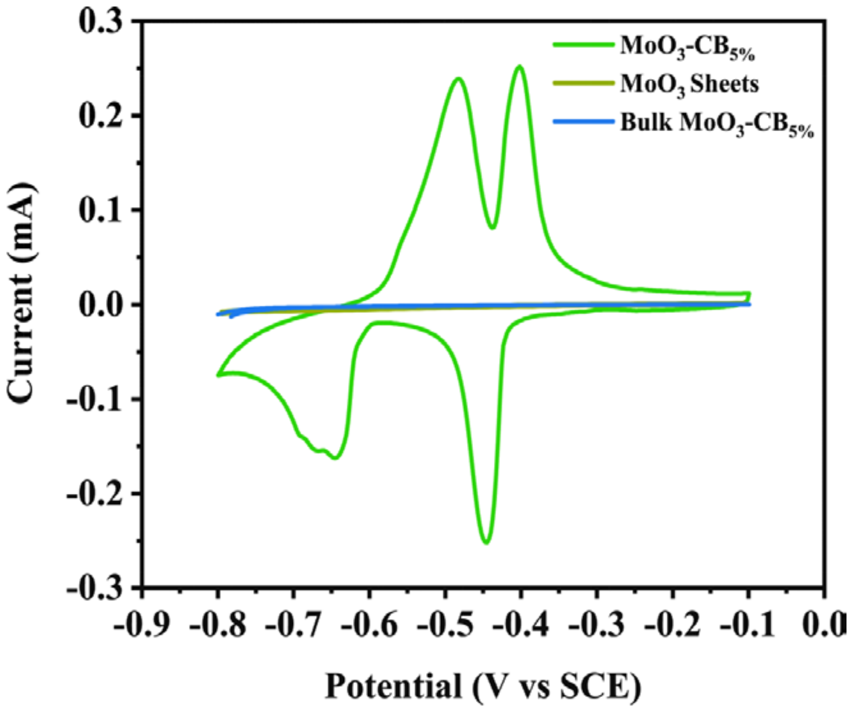

Figure S6: CV measurement comparing the performance of exfoliated MoO_3, the composite of exfoliated MoO_3 with 5 wt % CB and the composite of bulk MoO_3 with 5 wt % CB.

Table S1: Comparison of HSP values of 2-butanone and MoO_3.

Hansen solubility parameters	MoO_3 [1]	2-Butanone
Range of δ_D ($MPa^{1/2}$)	17.8	16
Range of δ_P ($MPa^{1/2}$)	8	9
Range of δ_H ($MPa^{1/2}$)	6.5	5.1

Table S2: Comparison of the specific capacitance of various MoO_3 structures and composites.

Material	Specific capacitance ($F \cdot g^{-1}$)	Reference
MoO_3 nanoparticles distributed uniformly in carbon matrix	179 @ 50 mA/g	[2]
MoO_3 nanowires	100 @ 200 mA/g	[3]
MoO_3 nanorod	30 @ 5 mV/s	[4]
Polypyrrole-coated α-MoO_3	125 @ 100 mA/g	[5]
ZnO@MoO_3 core/shell nanocables	236 @ 5 mV/s	[6]
MoO_3–MWCNT nanocomposites	210 @ 5 mV/s	[7]
**MoO_3-carbon black**	**201 @ 50 mV/s**	**This work**

References

1. Alsaif, M. M. Y. A.; Field, M. R.; Daeneke, T.; Chrimes, A. F.; Zhang, W.; Carey, B. J.; Berean, K. J.; Walia, S.; van Embden, J.; Zhang, B.; Latham, K.; Kalantar-zadeh, K.; Ou, J. Z. *ACS Appl. Mater. Interfaces* **2016,** *8,* 3482–3493. doi:10.1021/acsami.5b12076
2. Tao, T.; Chen, Q. Y.; Hu, H. P.; Chen, Y. *Mater. Lett.* **2012,** *66,* 102–105. doi:10.1016/j.matlet.2011.08.028
3. Liang, R.; Cao, H.; Qian, D. *Chem. Commun.* **2011,** *47,* 10305–10307. doi:10.1039/c1cc14030b
4. Shakir, I.; Shahid, M.; Yang, H. W.; Kang, D. J. *Electrochim. Acta* **2010,** *56,* 376–380. doi:10.1016/j.electacta.2010.09.028
5. Liu, Y.; Zhang, B.; Yang, Y.; Chang, Z.; Wen, Z.; Wu, Y. *J. Mater. Chem. A* **2013,** *43,* 13582–13587. doi:10.1039/c3ta12902k
6. Li, G. R.; Wang, Z. L.; Zheng, F. L.; Ou, Y. N.; Tong, Y. X. *J. Mater. Chem.* **2011,** *21,* 4217–4221. doi:10.1039/c0jm03500a
7. Shakir, I.; Shahid, M.; Cherevko, S.; Chung, C. H.; Kang, D. J. *Electrochim. Acta* **2011,** *58,* 76–80. doi:10.1016/j.electacta.2011.08.076
